# Knockdown *ATG4C* inhibits gliomas progression and promotes temozolomide chemosensitivity by suppressing autophagic flux

**DOI:** 10.1186/s13046-019-1287-8

**Published:** 2019-07-10

**Authors:** Zhi-peng Wen, Wen-jing Zeng, Yan-hong Chen, He Li, Jie-ya Wang, Quan Cheng, Jing Yu, Hong-hao Zhou, Zheng-zheng Liu, Jian Xiao, Xiao-ping Chen

**Affiliations:** 1 Department of Clinical Pharmacology, Xiangya Hospital, Central South University, Changsha, Hunan 410008 People’s Republic of China; 2 Institute of Clinical Pharmacology, Central South University; Hunan Key Laboratory of Pharmacogenetics, Changsha, Hunan 410078 People’s Republic of China; 3 National Clinical Research Center for Geriatric Disorders, Xiangya Hospital, Central South University, Changsha, Hunan 410008 People’s Republic of China; 4 Medical Genetic Institute of Henan Province, Henan Provincial Key Laboratory of Genetic Diseases and Functional Genomics, Henan Provincial People’s Hospital, People’s Hospital of Zhengzhou University, Zhengzhou, Henan, Hunan People’s Republic of China; 5 Department of Neurosurgery, Xiangya Hospital, Central South University, Changsha, Hunan 410008 People’s Republic of China; 6 Department of Oncology, Xiangya Hospital, Central South University, Changsha, 410008 Hunan province China; 7 Department of Pharmacy, Xiangya Hospital, Central South University, Changsha, Hunan 410008 People’s Republic of China

**Keywords:** Glioma, Glioblastoma, ATG4C, Autophagy, Temozolomide

## Abstract

**Background:**

Gliomas are the most common primary tumors in central nervous system. Despite advances in diagnosis and therapy, the prognosis of glioma remains gloomy. Autophagy is a cellular catabolic process that degrades proteins and damaged organelles, which is implicated in tumorigenesis and tumor progression. Autophagy related 4C cysteine peptidase (*ATG4C*) is an autophagy regulator responsible for cleaving of pro-LC3 and delipidation of LC3 II. This study was designed to investigate the role of ATG4C in glioma progression and temozolomide (TMZ) chemosensitivity.

**Methods:**

The association between *ATG4C* mRNA expression and prognosis of gliomas patients was analyzed using the TCGA datasets. The role of *ATG4C* in proliferation, apoptosis, autophagy, and TMZ chemosensitivity were investigated by silencing ATG4C in vivo. Ectopic xenograft nude mice model was established to investigate the effects of ATG4C on glioma growth in vivo.

**Results:**

The median overall survival (OS) time of patients with higher ATG4C expression was significantly reduced (HR: 1.48, *p* = 9.91 × 10^− 7^). *ATG4C* mRNA expression was evidently increased with the rising of glioma grade (*p =* 2.97 × 10^− 8^). Knockdown ATG4C suppressed glioma cells proliferation by inducing cell cycle arrest at G1 phase. ATG4C depletion suppressed autophagy and triggered apoptosis through ROS accumulation. Depletion of ATG4C suppressed TMZ-activated autophagy and promoted sensitivity of glioma cells to TMZ. Additionally, ATG4C knockdown suppressed the growth of glioma remarkably in nude mice.

**Conclusion:**

ATG4C is a potential prognostic predictor for glioma patient. Targeting ATG4C may provide promising therapy strategies for gliomas treatment.

**Electronic supplementary material:**

The online version of this article (10.1186/s13046-019-1287-8) contains supplementary material, which is available to authorized users.

## Background

Gliomas are the most common primary malignant tumors of central nervous system (CNS) in adults [1, 2]. According to the morphopathology features of tumor cells, gliomas are mainly classified into subtypes including astrocytoma, oligodendroglioma, ependymoma and choroid plexus papilloma [3, 4]. Based on pathological evaluation, gliomas are categorized as Grade I to IV, and higher grade indicates worse prognosis [3, 5]. The median survival time of patients with low-grade glioma (LGG, Grade II and III) is 3 to 10 years, compared with 1.5 years for patients with Grade IV (Glioblastoma) [6–10]. Temozolomide (TMZ) is considered to be the most effective drug for the treatment of glioblastoma, which can prolong the survival of glioblastoma patients by 2 to 5 months [9, 11–13]. Several molecular biomarkers, such as *IDH* mutation, 1p/19q co-deletion and *MGMT* promoter methylation, are shown to predict prognosis and/or drug responses for gliomas [3, 9, 13]. However, the overall prognosis of gliomas has not been improved effectively.

Macroautophagy (Autophagy) is an evolutionally conserved dynamic catabolic process that degrades proteins and damaged organelles [14, 15]. The role of autophagy in tumorigenesis and progression is complicated. On the one hand, autophagy deficiency promotes tumorigenesis; on the other hand, autophagy is essential for the progression of tumor [16–21]. Additionally, recent studies have suggested that TMZ-induced autophagy sustained the survival of glioblastoma cells, thereby contributing to drug resistance and recurrence [10, 22]. Blocking autophagy by inhibitor can significantly enhance TMZ cytotoxicity [23–25]. Whereas, non-specific autophagy inhibition may bring unexpected safety problems, which limits its clinical application [10, 26, 27].

The Cancer Genome Atlas (TCGA) project, aimed to depict profile of cancer genome, is widely used in tumor research. Based on TCGA data mining, we observed that expression level of autophagy related 4C cysteine peptidase (ATG4C), a member of ATG4 family, is deeply associated with prognosis of glioma patients. ATG4C is a cysteine peptidase responsible for lipidation and delipidation of LC3 during autophagy [28, 29]. Previous study has shown that ATG4C is involved in the maintenance of stem-like phenotype in breast cancer cells [30]. However, the role of ATG4C in gliomas progression and TMZ chemosensitivity remains unclear.

In this study, we tried to determine whether interference with ATG4C can affect autophagy activity and TMZ sensitivity in glioma cell lines, as well as its role in disease progression of glioma both in vitro and in vivo.

## Methods

### Patients samples

Glioma was diagnosed according to the 2016 WHO Classification of Tumors of the Central Nervous System. The glioma tissues and clinical data were obtained from patients who underwent surgery between 2015 June and 2018 January at Xiangya Hospital of Central South University. The informed consents were provided to all patients or their family members. This study was approved by Central South University Xiangya Hospital Medical Ethics Committee.

### Bioinformatic data mining

The integrated gliomas expression profile data and corresponding clinical data were downloaded from the TCGA database (http://www.cbioportal.org/study.do?cancer_study_id=lgggbm_tcga_pub). Clinical characteristics of the patients were shown in Table 1.

**Table 1:** Baseline clinical characteristics of patients

Clinical characteristic	Prognosis analysis in gliomas	Prognosis analysis in LGG	Prognosis analysis in GBM
NO. of patients	975	414	561
Age (years)			
≥55	546	326	220
<55	429	88	341
Gender			
Male	573	227	346
Female	402	187	215
WHO grade			
II	198	198	0
III	216	216	0
IV	561	0	561
IDH status			
Wild	483	77	
Mutant	372	337	
Undefined	120	2	
MGMT status			
Unmethylated	280		211
Methylated	514		169
Undefined	181		181

### Cell culture

The human glioma cell line U87-MG was purchased from Cell Bank of Chinese Academy, Shanghai Institute of Biochemistry and Cell Biology, China Academy of Science. Human glioma cell line T98G was kindly provided by Dr. Lv from Jiangxi Cancer Hospital, China. The cell lines were identified using short tandem repeat (STR) markers. Cells were cultured in Dulbecco’s modified Eagle’s medium (DMEM) (Gibco, CA, USA) supplemented with 10% fetal bovine serum (FBS) (Gibco, CA, USA) in a humiliated incubator containing 5% CO_2_ at 37 °C.

### Cell transfection

Three different small interfering RNA (siRNA) against *ATG4C* and negative control siRNA were synthesized by RiboBio (Guangzhou, China). Exponentially growing cells were seeded in 6 well plates and cultured overnight. The plated cell at 60–70% confluence was transfected with indicated siRNAs at a final concentration of 50 nM using Lipofectamine RNAiMAX reagent (Invitrogen, CA, USA) following the manufacturer’s instruction. The plasmid of RFP-GFP-LC3B was kindly provided by Professor Cheng, Central South University, China. Cells were transfected with 1.0 μg plasmid per well of a 6-well plate using Lipofectamine 3000 reagent (Invitrogen, CA, USA) according to the manufacturer’s instruction. Six hours after transfection, the medium was replaced with fresh medium containing 10% FBS. The transfected cells were collected for further analysis.

### Lentivirus infection and stable cell lines establishment

The knockdown lentivirus vector of *ATG4C* (LV-ATG4C-shRNA) and the lentivirus vector carrying scramble sequences (LV-NC-shRNA) were purchased from Genechem (Shanghai, China). All the lentivirus vectors were verified by DNA sequencing. After seeded in 6-well plates for 24 h, cells were infected with ATG4C sh-RNA (sh-ATG4C) or negative control sh-RNA (sh-NC) at a multiplicity of infection (MOI) of 10 in the Opti-MEM medium. The culture medium was replaced with DMEM containing 10% serum 12 h after, and cells were continually cultured in incubator at 37 °C with 5% CO_2_. To establish ATG4C stable knockdown cell lines, infected cells were treated with puromycin (2 μg/ml for U87-MG, 1 μg/ml for T98G) for 7 days. The knockdown efficiency was evaluated by Western blot analysis.

### Total RNA extraction and real-time qPCR

Total RNA was extracted from tissues or cells using RNAiso Plus reagent (Takara, China) and then reverse-transcribed to complementary DNA (cDNA) by PrimeScript RT Master Mix (Takara, China) according to the manufacturer’s instruction. Real-time PCR was performed with LightCycler 480 type II instrument (Roche, Switzerland). PCR were conducted using the following cycling parameters: pre-denaturation at 95 °C for 30 s, followed by 45 cycles of two step: 95 °C for 5 s and 60 °C for 20 s. *GAPDH* was selected to normalize genes expression levels using the ΔΔCt method. Primers used in this study were shown as Additional file 5: Table S1 (Ribobio, China).

### Western blot analysis

The whole-cell lysates were produced by RIPA buffer containing PMSF. The protein concentrations were quantified using BCA Protein Assay kit (Beyotime, China). Equal amounts of proteins were added to SDS-PAGE and then transferred onto PVDF membrane. Blots were blocked in TBST buffer containing 5% (m/v) nonfat milk at room temperature for 2 h and then incubated at 4 °C overnight with primary antibodies as follows: GAPDH (Protein Tech), ATG4C (Abcam), LC3 (Cell Signaling Technology), P62 (Cell Signaling Technology), p21 (Ab clonal), p53 (AB clonal), Cyclin E (Cell Signaling Technology), Bcl-2 (Protein Tech), BAX (Protein Tech) and PARP (Protein Tech). After washing with TBST buffer, the membranes were probed with secondary antibodies (Invitrogen, USA) at room temperature for 1 h. Immunoreactive binding was detected with BeyoECL star kit (Beyotime, China) using Bio-Rad ChemiDoc XRS imaging system (Bio-Rad, USA).

### Cell proliferation and viability assay

Cell proliferation and viability were detected by MTS. Briefly, cells were seeded in 96-well at a density of 1000 cells/well. After culturing for indicated time, the medium was replaced with 100 μL DMEM containing 10 μL MTS (Promega, CA, USA) and incubated at 37 °C for 1 h. Then, the optical density (OD) values were detected at 490 nm using multi-well spectrophotometer. The survival rate was calculated as AT_490_/CT_490_ × 100% (AT_490_ = Absorbance value of the experimental group at 490 nm; CT_490_ = Absorbance value of the control group at 490 nm). IC_50_ values were derived by GraphPad Prism 5 software through plotting the survival rates on a logarithmic curve.

### Colony forming assay

After transfection, cells were dissociated and seeded in 3.5 cm dishes at a destiny of 1.0 × 10^3^ cells/well and then cultured for 14 days. The cells were fixed with 4% (v/v) formaldehyde for 15 min, then stained with 0.1% (w/v) crystal violet for 10 min. After washing with PBS for three times, the number of colonies was counted.

### Cell cycle analysis

Briefly, cells were harvested and fixed overnight in a freezer at − 20 °C with precooled 70% ethanol, then centrifuged at 1000 g for 5 min. The pellet was resuspended with 100 μL PBS containing 2 μL of RNase A (10 mg/mL), then incubated at 37 °C for 30 min. After that, 400 uL of PBS including 20 uL of PI (1 mg/mL) and 0.4 μL of Triton-X were added and incubated at 37 °C for 30 min. The cell cycle distribution was analyzed flow cytometer (Beckman Coulter, USA).

### Cell apoptosis assay

Cells apoptosis was evaluated by Annevin V-FITC/PI and Hoechst 33342 staining. For Annevin V-FITC/PI staining, cells were dissociated by trypsin without EDTA and centrifuged at 1000 g for 5 min. The pellet was resuspended with staining solution containing 5 μL Annexin V-FITC and 10 μL PI, then incubated at room temperature for 20 min in dark. The apoptosis was analyzed by flow cytometer following manufacture’s instruction (Beyotime, China). For Hoechst 33342 staining, cells were fixed by 4% (v/v) formaldehyde for 10 min and washed with PBS, then staining buffer with Hoechst 33342 were added. The apoptotic cells were observed under fluorescence microscope. For Caspase-3/6/9 activity assay, cells were harvested and centrifuged at 1000 g for 5 min, and then incubated with lysis buffer for 15 min on ice. The lysate was centrifuged at 12000 g for 15 min. Then the supernatant was detected for the caspase-3/6/9 activity by multi-well spectrophotometer, according to the manufacture’s instruction (Beyotime, China).

### Determination of ROS in cultured cells

After transfection, T98G cells were harvested and centrifuged at 1000 g for 5 min. The pellet was resuspended with DMEM containing DCFH-DA (10 μM) and incubated for 20 min. After that, the cell suspension was centrifuged at 1000 g for 5 min and resuspended with 400 μL PBS, then analyzed by flow cytometer.

### Nude mice xenograft study

This study was performed in compliance with the guidelines of the Ethics Committee of Institutional Animal Care and Use in Central South University. The BALB/C male nude mice were obtained from Hunan SLAC Co., Ltd. The mice were housed in a specific pathogen-free facility at Department of Laboratory, Central South University. The ATG4C stable knockdown U87-MG cells were harvested and resuspended in PBS containing Matrigel matrix (BD, USA) at a density of 1 × 10^8^ cells/ml. Then mice were injected subcutaneously with 100 μL of cells suspension (1 × 10^7^ cells/mouse). Tumor volume and mice weight were measured after implanted for indicated time. Tumor volume (mm^3^) = (L × W^2^)/2, where L and W (L > W) are the tumor’s length and width. At the end point, tumor tissues were weighted and fixed in paraffin for further analysis.

### Immunohistochemistry (IHC)

IHC was performed to determine the expression level of Ki67, LC3 and ATG4C in tumor tissue implanted in mice. Briefly, the formalin-fixed, paraffin-embedded samples were cut into 4 μm slices. Then, tissues sections were baked, dewaxed, hydrated and blocked. Afterwards, the sections were incubated with primary antibodies against Ki-67, LC3B and ATG4C. The following day, sections were incubated with secondary antibody at room temperature. After visualizing, sections were photographed using microscope.

### Statistical analysis

All statistical analysis was performed using SPSS software (version 16.0). Data were presented as the mean ± standard deviation. Statistical analysis between two groups was performed using *t*-test. One-way ANOVA with Tamhane *post-hoc* was used for comparisons of different groups. Comparisons of survival rate were analyzed using Kaplan-Meier method and log-rank test. Multivariate Cox proportional hazard models were used to analyze the effect of clinical variables on patients’ overall survival. *P*-value of < 0.05 was considered as statistically significant in this study.

## Results

### Higher *ATG4C* mRNA expression predicted poor prognosis for glioma patients

**Fig. 1:**
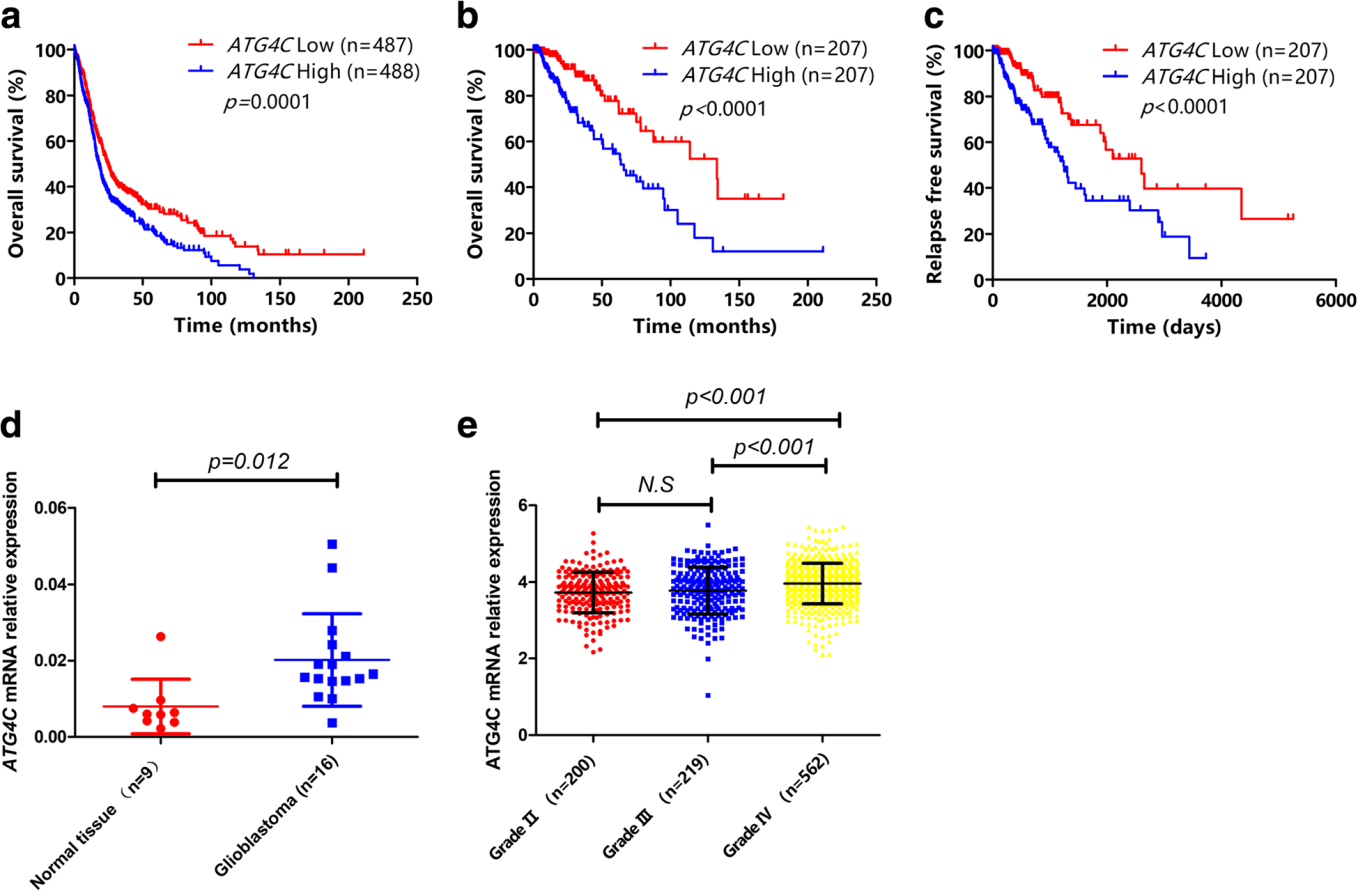
Kaplan-Meier survival analysis of glioma patients based on *ATG4C* mRNA expression levels. **a** Comparison of overall survival (OS) profiles between patients with *ATG4C* high and low expression levels in 975 glioma patients. **b** Comparison of overall survival (OS) profiles between patients with *ATG4C* high and low expression levels in 414 LGG patients. **c** Comparison of relapse free survival (RFS) time between patients with *ATG4C* high and low expression levels in 414 LGG patients. **d** Comparison of *ATG4C* mRNA expression between 16 glioblastoma tissues and 9 normal brain tissues. **e** Comparison of the mRNA expression of *ATG4C* in patients with different pathological grades of gliomas

In order to investigate the role of autophagy-related genes (ATGs) in prognosis of glioma patients, we developed an analysis flowchart (Additional file 1: Figure S1). The baseline clinical characteristics of glioma patients were shown in Table 1. We observed that the mRNA expression of 13 ATGs (*ATG3*, *ATG4A*, *ATG4C*, *ATG5*, *ATG7*, *ATG9B*, *ATG10*, *ATG12*, *ATG16L1*, *ATG2A*, *ATG2B*, *ATG9A* and *ATG16L2*) were closely associated with the OS of glioma patients (Fig. 1a and Additional file 2: Figure S2). To rule out the potential influence of WHO grade, *MGMT* promoter methylation, *IDH* mutation and other clinical variables on prognosis of patients, Cox proportional hazards regression was further performed. The results showed that mRNA expression of *ATG3* (HR = 0.63, 95% CI: 0.40–0.99, *p* = 0.05), *ATG4C* (HR = 1.54, 95% CI: 1.16–2.01, *p* = 3 × 10^− 3^) and *ATG5* (HR = 0.61, 95% CI: 0.43–0.86, *p* = 5 × 10^− 3^) were independent OS prognostic factors in gliomas patients (Table 2). Given the significant influence of pathological grade on patient prognosis, these findings were further validated in low-grade glioma (LGG) patients and glioblastoma patients (GBM), respectively. We observed that only *ATG4C* mRNA expression was significantly associated with OS in LGG patients (Fig. 1b and Additional file 3: Figure S3). Moreover, the median relapse-free survival (RFS) time of LGG patients with low *ATG4C* mRNA expression was significantly longer than those with high expression (Fig. 1c). However, no significant association was observed between ATGs mRNA expression and OS in GBM patients (Additional file 3: Figure S3).

**Table 2:** Cox proportional hazards regression analysis of factors associated with overall survival of glioma patients

Variable	Univariate analysis		Multivariate analysis	
	HR (95% CI)	*p* value	HR (95% CI)	*p* value
*MGMT*	0.37 (0.30–0.46)	6.81 × 10^−19^	0.98 (0.75–1.28)	0.88
IDH mutation vs wild	0.13 (0.10–0.17)	6.05 × 10^−51^	0.40 (0.26–0.63	6.40 × 10^− 5^
Grade III vs Grade II	3.20 (1.93–5.29)	5.86 × 10^−6^	2.44 (1.44–4.12)	9.34 × 10^−4^
Grade IV vs Grade II	14.28 (9.14–22.32)	1.88 × 10^− 31^	5.02 (2.70–9.36)	3.71 × 10^−6^
Age	1.06 (1.05–1.06)	7.91 × 10^−58^	1.05 (1.03–1.06)	3.85 × 10^− 15^
Gender	1.13 (0.94–1.35)	0.19	1.51 (1.17–1.93)	0.014
*ATG2A* mRNA	0.41 (0.35–0.48)	2.95 × 10^− 30^	1.17 (0.79–1.73)	0.43
*ATG2B* mRNA	0.47 (0.40–0.54)	4.50 × 10^−24^	0.98 (0.75–1.27)	0.86
*ATG3* mRNA	1.42 (1.10–1.84)	0.008	0.63 (0.40–0.99)	0.05
*ATG4A* mRNA	2.37 (2.02–2.79)	1.04 × 10^− 25^	0.90 (0.64–1.28)	0.56
*ATG4C* mRNA	1.48 (1.27–1.73)	9.91 × 10^− 7^	1.54 (1.16–2.01)	3 × 10^− 3^
*ATG5* mRNA	1.74 (1.43–2.13)	4.32 × 10^− 8^	0.61 (0.43–0.86)	5 × 10^−3^
*ATG7* mRNA	2.32 (1.89–2.86)	1.66 × 10^− 15^	1.36 (0.94–1.98)	0.1
*ATG9A* mRNA	0.42 (0.34–0.51)	7.05 × 10^− 17^	0.74 (0.49–1.13)	0.16
*ATG9B* mRNA	1.44 (1.33–1.57)	1.57 × 10^− 18^	0.97 (0.88–1.07)	0.52
*ATG10* mRNA	2.18 (1.86–2.55)	1.08 × 10^− 21^	1.11 (0.82–1.41)	0.50
*ATG12* mRNA	2.05 (1.62–2.61)	4.59 × 10^− 9^	0.998 (0.66–1.52)	0.99
*ATG16L1* mRNA	3.17 (2.53–3.97)	8.53 × 10^−24^	1.01 (0.77–1.09)	0.96
*ATG16L2* mRNA	0.78 (0.70–0.88)	7.22 × 10^−5^	0.92 (0.77–1.09)	0.34

### mRNA expression of *ATG4C* was increased with pathological grades in glioma patients

To make clear whether ATG4C was differentially expressed between glioblastoma and normal brain tissues, mRNA levels of *ATG4C* was detected in 16 glioblastoma and 9 normal brain tissues. As compared with normal brain tissues, the expression of *ATG4C* mRNA was significantly higher in glioblastoma tissues (Fig. 1d). We then compared the expression of *ATG4C* mRNA in different grades of gliomas using TCGA database. As shown in Fig. 1e, *ATG4C* mRNA expression was significantly increased with the increase in glioma grade. When the patients were divided into two groups according to *ATG4C* mRNA expression levels, we observed that patients with higher *ATG4C* mRNA expression were more likely to develop advanced grades glioma (Table 3).

**Table 3:** Comparison of distribution of clinical factors between glioma patients with high and low ATG4C mRNA expression

Clinical characteristic	NO. of patients		*p* value
	ATG4C low expression	ATG4C high expression	
NO. of cases	487	488	
Age (years)			
≥ 55	297 (60.99%)	249 (51.02%)	0.002
< 55	190 (39.01%)	239 (48.98%)	
Gender			
Male	279 (57.29%)	294 (60.25%)	0.348
Female	208 (42.71%)	194 (39.75%)	
WHO grade			
II	121 (24.85%)	77 (15.78%)	0.000
III	120 (24.64%)	96 (19.67%)	
IV	246 (50.51%)	315 (64.55%)	
IDH status			
Wild	185 (37.99%)	130 (26.64%)	0.000
Mutant	242 (49.69%)	298 (61.07%)	
Undefined	60 (12.32%)	60 (12.30%)	
MGMT status			
Unmethylated	110 (22.59%)	224 (45.90%)	0.000
Methylated	290 (59.55%)	170 (34.84%)	
Undefined	87 (17.86%)	94 (19.26%)	

### Knockdown of ATG4C suppressed glioma cells proliferation

**Fig. 2:**
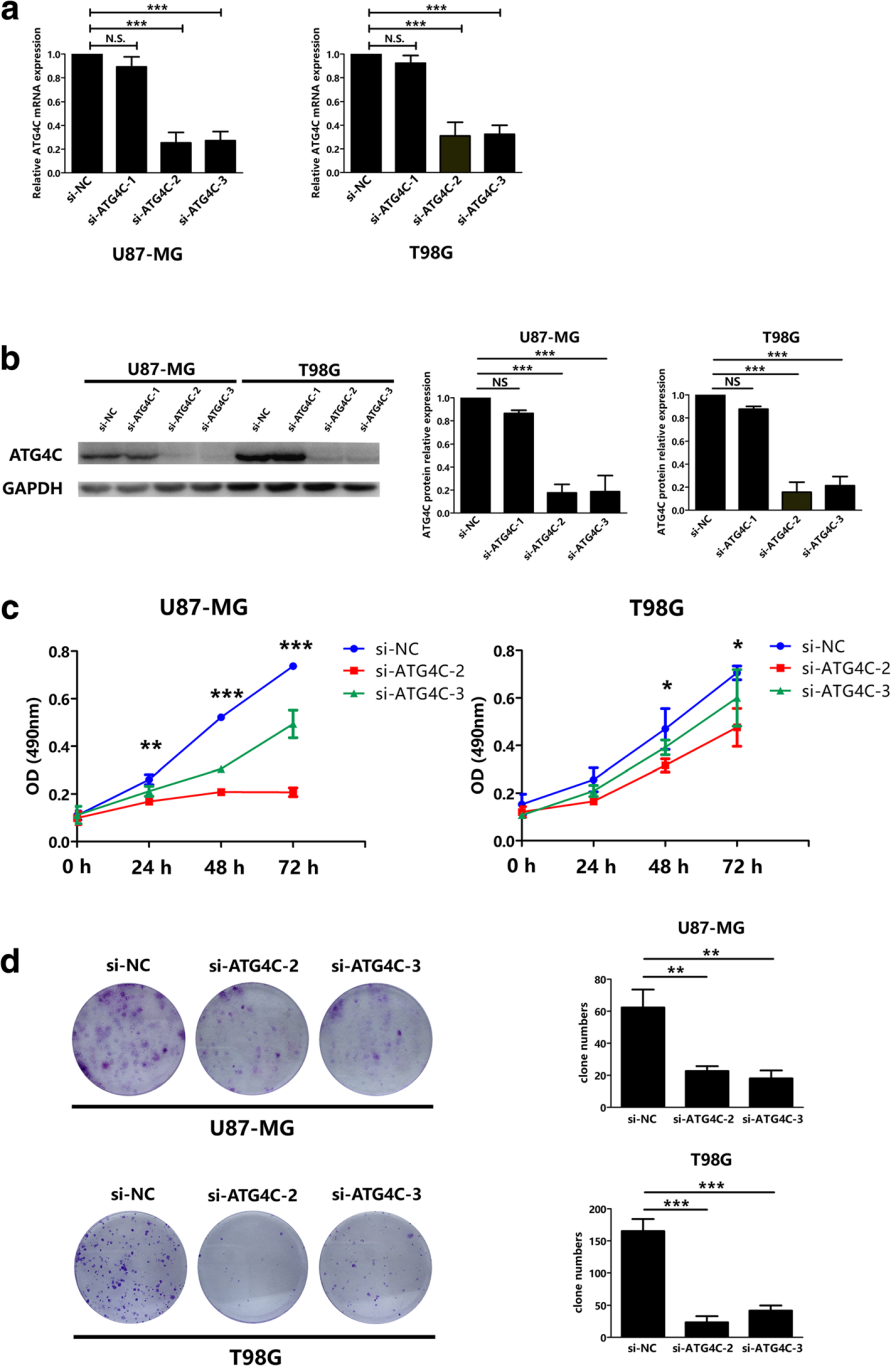
Knockdown of ATG4C inhibited the proliferation of glioma cells. **a-b** ATG4C siRNAs interference efficiency was tested at both mRNA and protein levels. **c** ATG4C knockdown by si-ATG4C transfection suppressed the proliferation of glioma cells in a time-dependent manner. **d** ATG4C knockdown by si-ATG4C transfection impaired clone formation ability of glioma cells. Left panel: representative crystal violet staining images; right panel: quantitative analysis of colony numbers. *p < 0.05, **p < 0.01, ***p < 0.001, NS: not significant

The above results indicated that *ATG4C* mRNA expression could predict the prognosis of glioma patients, implying that *ATG4C* might be involved in glioma progression and/or therapeutic response. The MTS assay and colony formation were performed to evaluate the role of ATG4C on glioma proliferation. The interference efficiency of siRNAs was validated at both mRNA and protein levels (Fig. 2a and b). Compared with control group, knockdown of ATG4C by si-RNA transfection significantly suppressed the proliferation of glioma cells indicated by proliferation curves (Fig. 2c). Results from colony-formation assay also showed that knockdown of ATG4C by si-RNA transfection reduced the number of colonies remarkably (Fig. 2d).

### ATG4C ablation interfered with cell cycle and the expression of cell cycle related proteins in glioma cells

**Fig. 3:**
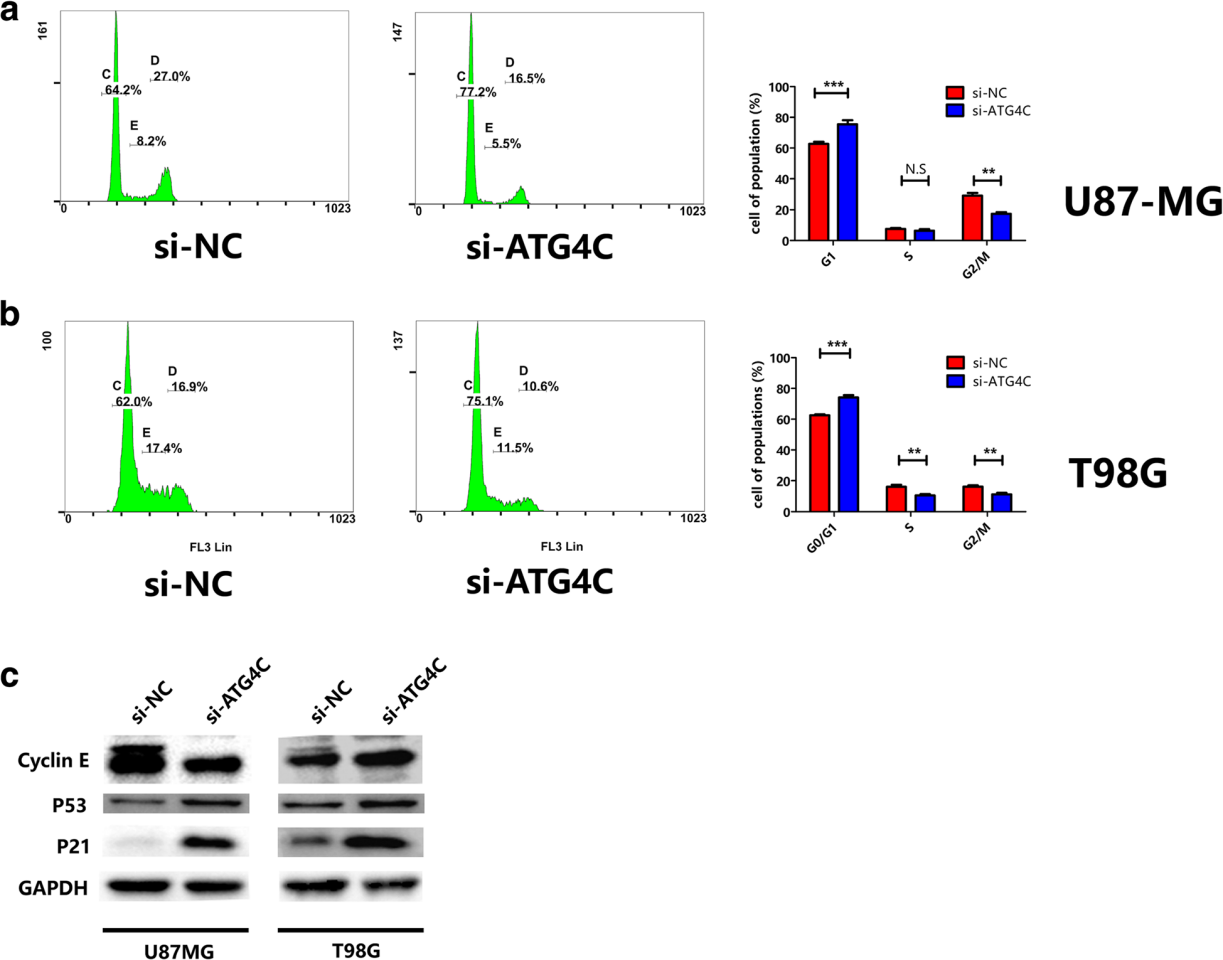
Influence of ATG4C knockdown on cell cycle and the expression of cell-cycle related proteins in glioma cells. **a-b** Changes in proportion of cells in G1 phase with ATG4C depletion. **c** Changes in protein expression of p21 and p53 by ATG4C depletion

The impaired proliferation of glioma cell by si-ATG4C might be a result, at least in part, from unorganized cell cycle. For this reason, we analyzed the cell cycle distribution of glioma cells after ATG4C silencing. Both U87-MG and T98G cells transfected with ATG4C siRNA were accumulated in G1 phase (Fig. 3a and b). Meanwhile, knockdown of ATG4C increased the protein expression of p21 and p53 while decreased the expression of Cyclin E (Fig. 3c).

### ATG4C knockdown suppressed autophagic flux in glioma cells

**Fig. 4:**
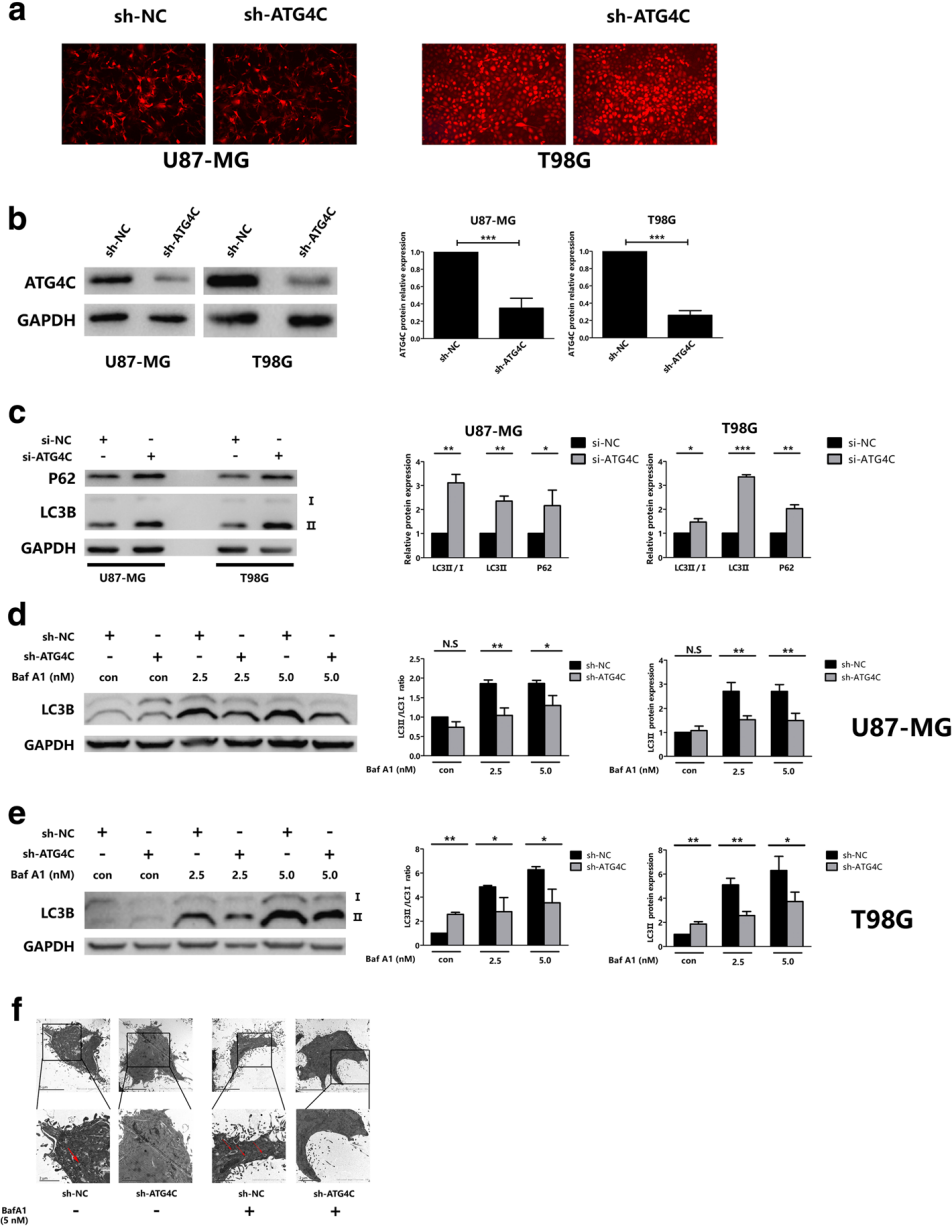
Knockdown of ATG4C suppressed autophagy and autophagic flux. **a**, **b** ATG4C stable knockdown glioma cells were established by lentivirus infection, and the interference efficiency was detected by Western blot analysis. **c** Protein expression of LC3-II and P62 in glioma cells transfected with si-ATG4C. **d**, **e** Knockdown of ATG4C on the expression of LC3-II in the presence of BafA1. **f** Transmission electron microscopy observation of cellular ultrastructure tin T98G cells by ATG4C depletion. Arrows indicate autophagic vacuoles. *p < 0.05, **p < 0.01, ***p < 0.001, NS: not significant

Previous studies have shown that ATG4C was implicated in the formation of autophagsome [31, 32]. However, the role of ATG4C in autophagy in glioma cells was unclear. ATG4C stable knockdown glioma cell lines were established (Fig. 4a and b). The LC3II/LC3-I ratio and expression of LC3-II and P62 are established indicators of autophagy. Intriguingly, we found that both of LC3-II and P62 protein levels were obviously increased in cells treated with si-ATG4C (Fig. 4c). In order to further confirm the role of ATG4C in autophagy, an autophagic flux assay was performed in ATG4C stable knockdown cells. Autophagic flux can be evaluated by LC3-II protein levels in the presence or absence of inhibitors of lysosomal degradation such as Bafilomycin A1 (BafA1). Our results showed that, in the presence of BafA1, knockdown of ATG4C reduced the protein expression of LC3-II remarkably, which suggested impaired autophagic flux (Fig. 4d and e). The analysis of transmission electron microscope (TEM) was performed to observe the influence of ATG4C on the formation of autophagy vacuoles in T98G cells. As shown in Fig. 4f, autophagic vacuoles was increased in T98G cells by BafA1 treatment. And in the presence of BafA1, knockdown of ATG4C reduced autophagic vacuoles.

### ATG4C depletion triggered apoptosis by increasing ROS

**Fig. 5:**
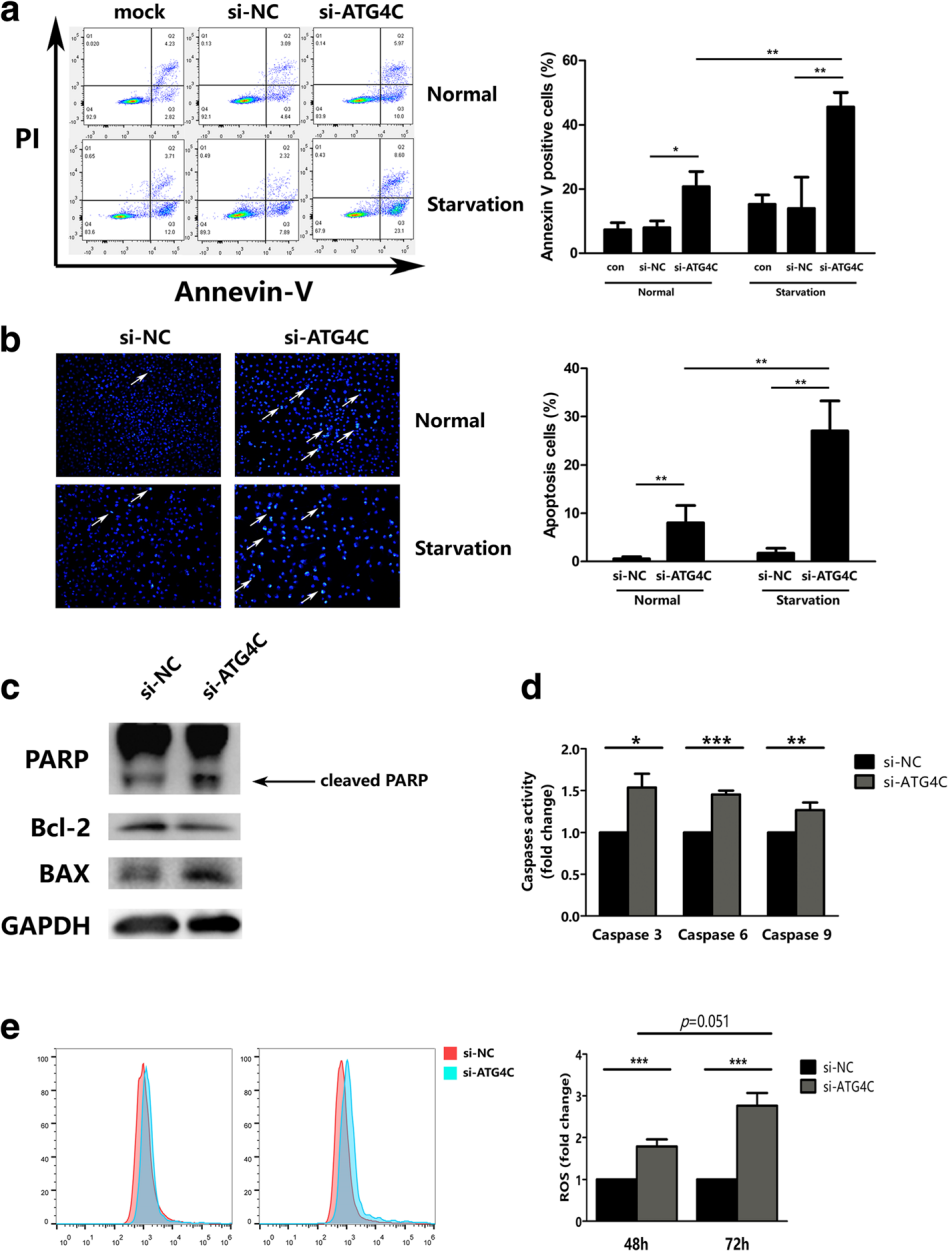
Knockdown of ATG4C promoted apoptosis and increased ROS accumulation in T98G cells. **a-b** The apoptosis in T98G cell transfected with si-ATG4C was detected by Annevin-V and Hoechst 33342 staining, respectively. **c** Changes in protein levels of cleaved-PARP, BAX and Bcl-2 expression in T98G cells transfected with ATG4C siRNAs. **d** Changes in Caspase-3/6/9 activity in T98G cells transfected with *ATG4C* siRNAs. **e** Changes in ROS levels in T98G cells transfected with *ATG4C* siRNAs. *p < 0.05, **p < 0.01, ***p < 0.001, NS: not significant

Beside immortal proliferation, decreased apoptosis is another important malignant biological behavior of gliomas. The effect of ATG4C on apoptosis was investigated in T98G using Annexin V-FITC/PI and Hoechst 33342 staining. As shown in Fig. 5a and b, knockdown of ATG4C significantly promoted apoptosis in T98G cells. And ATG4C depletion-induced apoptosis was further enhanced by starvation, a known factor for autophagy stimulation. Western blot analysis showed that ATG4C ablation remarkably increased the expression of pro-apoptosis proteins BAX and cleaved-PARP, while reduced the expression of the anti-apoptosis protein Bcl-2 (Fig. 5c). Consistently, ATG4C ablation significantly increased the activity of caspase-3/6/9 (Fig. 5d). Autophagy deficiency is reported to lead to an increase in ROS levels and then trigger apoptosis. For this reason, we further investigated the influence of ATG4C ablation on ROS production. As shown in Fig. 5e, ATG4C ablation increased the level of ROS remarkably in a time-dependent manner in T98G cells.

### ATG4C depletion increased the sensitivity of glioma cells to TMZ

**Fig. 6:**
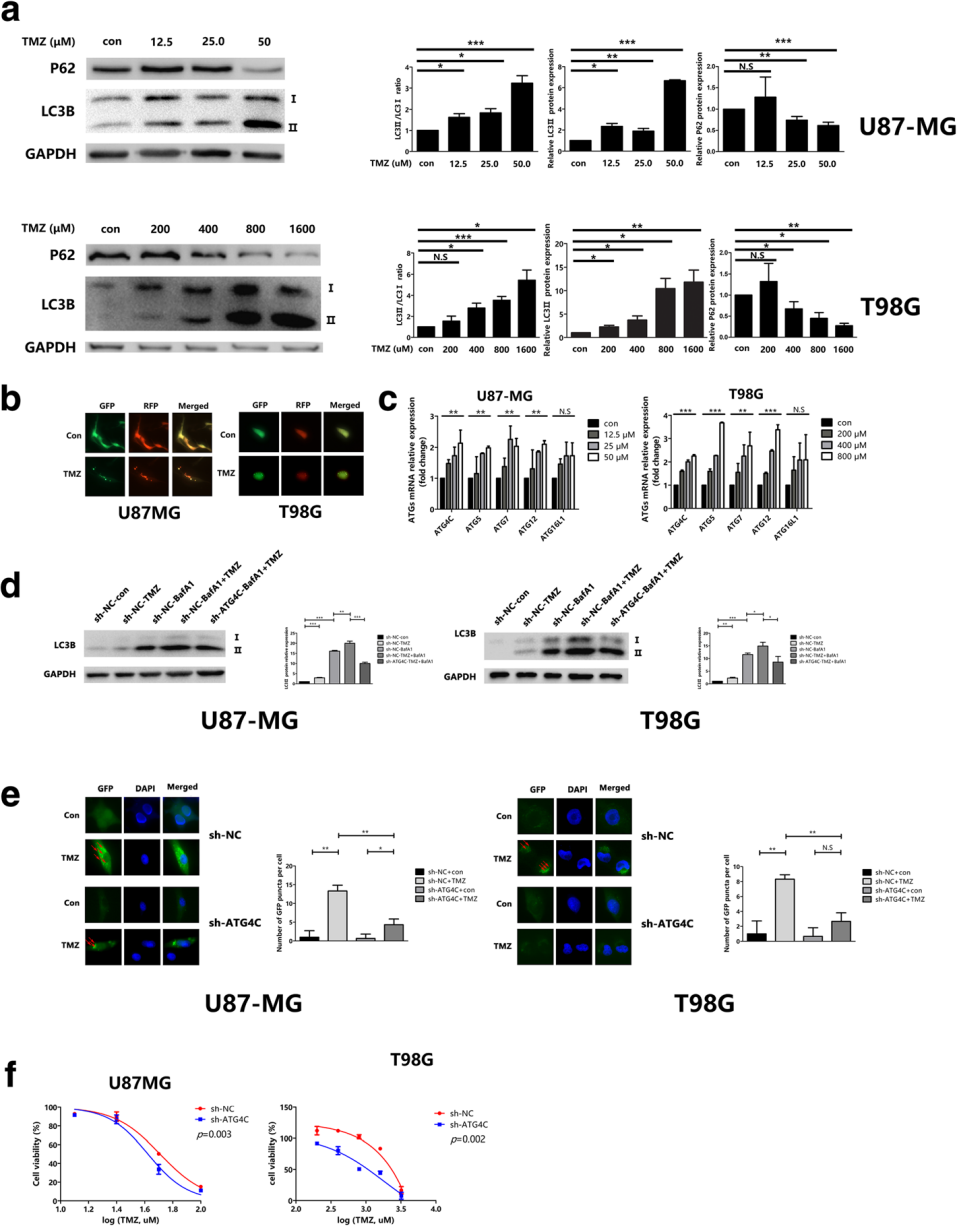
Knockdown of ATG4C enhanced TMZ cytotoxicity in glioma cells by suppressing autophagy. **a** Both LC3-II/LC3-I ratio and LC3-II expression were increased and P62 expression was decreased in glioma cells treated with TMZ. **b** The number of yellow puncta was elevated in glioma cells treated with TMZ. **c** The expression of ATGs mRNA were increased in glioma cells treated with TMZ. **d** Knockdown of ATG4C by sh-ATG4C decreased the expression of LC3-II in the presence of BafA1 in glioma cells treated with TMZ. **e** Knockdown of ATG4C by sh-ATG4C decreased the number of green puncta (red arrows) in glioma cells treated with TMZ. **f** Knockdown of ATG4C by sh-ATG4C increased TMZ cytotoxicity in glioma cells. *p < 0.05, **p < 0.01, ***p < 0.001, NS: not significant

Previous studies have shown that TMZ-induced autophagy sustained the survival of tumor cells, which contributed to treatment resistance. Blocking autophagy with inhibitors can significantly increase TMZ cytotoxicity in glioma cells. However, it is unknown whether ATG4C is involved in the TMZ-activated autophagy. To validate the effect of TMZ in activating autophagy in glioma cells, U87-MG and T98G cells were treated with series concentrations of TMZ (12.5, 25, and 50 μM for U87-MG cells, and 200, 400, 800, 1600 μM for T98G cells). Western blot analysis showed that TMZ increased the LC3-II/LC3-I ratio and LC3-II protein levels in a concentration-dependent manner in both cells (Fig. 6a). TMZ treatment also decreased the protein expression of P62 significantly (Fig. 6a). The autophagy activating effect of TMZ in glioma cells was further confirmed by an increase in RFP-GFP-LC3 puncta (Fig. 6b). Additionally, RT-qPCR analysis showed a significant increase in *ATG4C* mRNA levels with increasing TMZ concentrations (Fig. 6c). Moreover, we observed that ATG4C ablation blocked the accumulation of LC3II in the presence of BafA1 in the glioma cells treated with TMZ (Fig. 6d). Consistently, ATG4C depletion significantly decreased the increase of GFP-LC3 dots induced by TMZ (Fig. 6e). All these results suggested that ATG4C was involved in TMZ-induced autophagy. As shown in Fig. 6f, ATG4C ablation also decreased the IC_50_ of TMZ in both U87MG and T98G cells obviously (Fig. 6f).

### ATG4C knockdown inhibited the growth of xenograft tumor

**Fig. 7:**
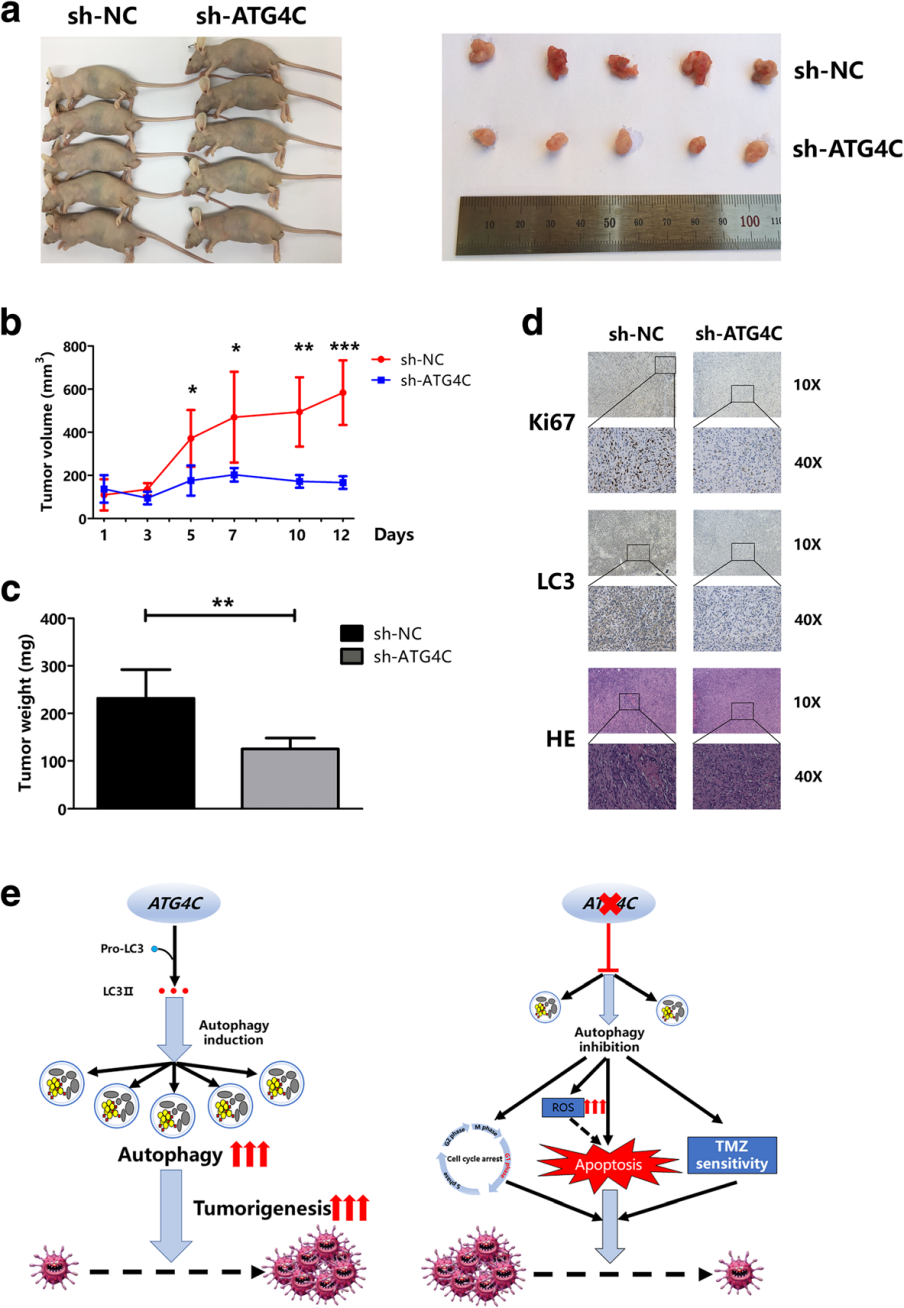
Knockdown of ATG4C by sh-ATG4C suppressed xenograft tumor and the graphic abstract. **a-b** The growth of glioma was restricted in nude mice by ATG4C knockdown. **c** Knockdown of ATG4C decreased the xenograft weights. **d** Depletion of ATG4C reduced the expression of Ki67, LC3 and ATG4C protein in xenograft. **e** Graph depicts the role of ATG4C in glioma progression and TMZ sensitivity

In order to investigate the effects of ATG4C on glioma growth in vivo, a xenograft nude mouse model was established. ATG4C stable knockdown U87-MG cells were implanted in nude mice and showed significantly slower growth than those in control group (Fig. 7a and b). Additionally, the tumors from mice injected with ATG4C depleted U87-MG cells was significantly smaller than those in control group (Fig. 7c). Moreover, ATG4C knockdown also remarkably impaired the proliferation of primary glioblastoma cells (Additionaly file 4: Figure S4). Immunohistochemical (IHC) staining was performed to detect the expression of Ki67, LC3 and ATG4C in the xenograft. As shown in Fig. 7d, the expression of Ki67, LC3 and ATG4C were decreased significantly in tumor tissues from sh-ATG4C group.

## Discussion

In this study, we identified that the mRNA level of *ATG4C* was associated with worse prognosis in glioma patients. ATG4C levels was evidently elevated with the rising of glioma grade. Knockdown of ATG4C markedly suppressed the growth of glioma and promoted apoptosis in glioma cells, which was accompanied by increased ROS production. Additionally, cytotoxicity of TMZ was increased while the TMZ-induced autophagy was reduced in ATG4C depleted glioma cells.

More and more molecular biomarkers are identified and applied to improve the diagnosis and treatment of gliomas. However, the prognosis of the disease still remains gloomy [3, 33–37]. And also, it is of desperate need to find promising therapeutic targets for glioma. In this study, we observed that ATG4C was differently expressed between normal brain and glioblastoma tissues, and its expression was increased with the grade of glioma. And also, we observed that ATG4C level was an independent predictor for OS and RFS in LGG patients (Additional files 6 and 7: Tables S2 and S3). To make clear whether ATG4C acts through affecting cell growth in gliomas, we observed its role in proliferation of gliomas cells. We demonstrated that knockdown ATG4C remarkably suppressed the proliferation of glioma cell lines by inducing cell cycle arrest at G1 phase. It is obvious that cell cycle transitions in eukaryotic cells are regulated by cyclin-dependent kinases (CDKs)-cyclin complexes [38]. The CDK inhibitor P21 is an important regulator of cell growth in mammalian cells [39] as well as a universal cell-cycle inhibition controlled by P53 [40, 41]. In our current works, we observed that knockdown ATG4C induced a decrease in Cyclin E and an increase in P21 and P53 expression, which indicate obvious cell cycle arrest caused by ATG4C ablation. Additionally, we observed that knockdown ATG4C could restrain the proliferation of primarily cultured glioblastoma cells from patients (Additional file 3: Figure S3). A large body of evidence suggests that inhibition of the activity of other ATG4 family members, including ATG4A, ATG4B and ATG4D, could suppress tumor progression and enhance chemosensitivity or the efficacy of radiotherapy [42–49]. These findings consistently implied that the ATG4 family played an important role in tumorigenesis and cancer therapy. Development of ATG4C specific inhibitors may bring new promising strategy for glioma treatment.

The role of ATG4C in autophagy in glioma cells is not clear. Previous researches suggested that the miR-376 mediated ATG4C silencing could suppress autophagy in breast cancer cells and hepatocarcinoma cells [31, 32]. Recently, there was data implied that ATG4C had a limited role in autophagy [50]. In the current work, we observed that LC3-II expression was significantly reduced in the presence of BafA1 in sh-ATG4C cells, indicating impaired autophagic flux. Results from transmission electron microscope (TEM) analysis also observed a decrease in autophagic vacuoles by ATG4C silencing, in the presence of BafA1. However, we observed concomitantly increased protein expression of LC3-II and P62 in glioma cells transfected with si-ATG4C. It seems to be intriguing. According to the functions of ATG4 family members in LC3 processing, ATG4C is putative to cleavage of pro-LC3 and delipidation of LC 3 II from the membrane of the autophagsome through the cysteine peptidase activity [28, 31, 32]. Of course, the possible role of ATG4C in LC3-II degradation at late stage of autophagy could not be excluded in our study.

Autophagy is essential for the removal of damaged mitochondria, which are primary sources of intracellular ROS [16]. Autophagy deficiency can lead to an increase in ROS, which contribute to the DNA damage, genomic instability and then trigger apoptosis [51–54]. In our study, we found that ROS levels were increased remarkably in T98G cells transfected with si-ATG4C for 48 h, and a significant increase in apoptosis was observed followed by si-ATG4C transfection for 72 h. These findings implied that ATG4C ablation could suppress autophagy in glioma cells and then trigger apoptosis by increasing ROS levels. However, ROS scavengers should be used to test whether ATG4C depletion-induced apoptosis was ROS-dependent. Moreover, ectopic xenograft nude mice model was used to establish the influence of ATG4C on glioma growth in vivo. Consistent with the in vivo findings, knockdown ATG4C restrained the proliferation of glioma with significantly decreased tumor volumes and weights. In tumor tissues of mice xenograft, the expression of both Ki67 and LC3 proteins were decreased in the sh-ATG4C group. These results indicated that ATG4C ablation may impaired the proliferation of glioma through suppressing autophagy in vivo.

TMZ is the first-line chemotherapeutic drug for glioma. The drug undergoes spontaneous hydrolysis to 5-(3-methyltriazen-1-yl) imidazole-4-carboxamide (MITC) and then forms O^6^-methylguanine (O^6^-MeG) in neutral pH. O^6^-MeG lead to DNA mispairs during DNA replication, and that results in DNA double strand breaks through futile cycles of mismatch repair system [55, 56]. In the treatment of glioma, cytotoxicity chemotherapeutics drugs including TMZ can induce autophagy, which may promote the development of treatment resistance [23–25, 57]. In our study, we observed for the first time that ATG4C is involved in TMZ-induced autophagy, and depletion of ATG4C significantly increased the sensitivity of U87-MG and T98G cells to TMZ, which are thought to be TMZ sensitivity and resistance cells, respectively [58]. Selective and specific cytotoxicity to tumor cells is expected during glioma chemotherapy, which can increase the anti-tumor activity of TMZ with less toxic effects. However, these findings need to be further validated in vivo. Given that ATG4C expression was increased in tumor tissues of glioma, a therapy targeting ATG4C may provide a promising strategy for gliomas treatment.

## Conclusion

Taken together, our results suggested that increased ATG4C expression was associated with worse prognosis in glioma patients. Knockdown of ATG4C suppressed glioma progression by inducing cell cycle arrest and promoting apoptosis of glioma cells possibly through increasing ROS production. Additionally, ATG4C ablation could promote TMZ cytotoxicity to glioma cells by inhibiting autophagy. Therefore, ATG4C might be potential target for the treatment of gliomas.

## Additional files

Additional file 1: **Figure S1.** Flow chart of procedures for screening of ATGs associated with outcomes of glioma patients. (TIF 90 kb)

Additional file 2: **Figure S2.** Survival analysis of glioma patients based on expression of ATGs. (a-m) Kaplan-Meier analysis for OS in gliomas patients. (TIF 2897 kb)

Additional file 3: **Figure S3.** Survival analysis of LGG based on expression of ATGs. (TIF 2249 kb)

Additional file 4: **Figure S4.** Knockdown of ATG4C suppressed cell growth in glioblastoma primary cells. a The expression of GFAP in glioblastoma primary cells. b ATG4C knockdown suppressed the proliferation of primarily cultured glioblastoma cells. (TIF 952 kb)

Additional file 5: **Table S1.** Sequences of primers used for RT-qPCR. (DOCX 14 kb)

Additional file 6: **Table S2.** Cox proportional hazards regression analysis for OS in LGG patients. (DOCX 17 kb)

Additional file 7: **Table S3.** Cox proportional hazards regression analysis for RFS in LGG patients. (DOCX 18 kb)

## Data Availability

All data generated or analyzed during this study are included in this published article. The TCGA data set can download from: http://www.cbioportal.org/study.do?cancer_study_id=lgggbm_tcga_pub.

## Abbreviations

ATG4C: Autophagy Related 4C Cysteine Peptidase
DMEM: Dulbecco’s Modified Eagle’s Medium
FBS: Fetal Bovine Serum
GBM: Glioblastoma
IHC: Immunohistochemistry
LGG: Low Grade Glioma
MITC: 5-(3-methyltriazen-1-yl) imidazole-4-carboxamide
O^6^-MeG: ROS: O^6^-methylguanine; Reactive Oxygen Species
OS: Overall Survival
qPCR: Quantitative polymerase chain reaction
RFS: Relapse-Fess Survival
TCGA: The Cancer Genome Atlas
TEM: Transmission electron microscope
TMZ: Temozolomide
WHO: World Health Organization
