# Supplementary figures and images for: Knockdown ATG4C inhibits gliomas progression and promotes temozolomide chemosensitivity by suppressing autophagic flux
Source: J Exp Clin Cancer Res. 2019 Jul 10;38:298. doi: 10.1186/s13046-019-1287-8 (PMC6617611; doi:10.1186/s13046-019-1287-8)

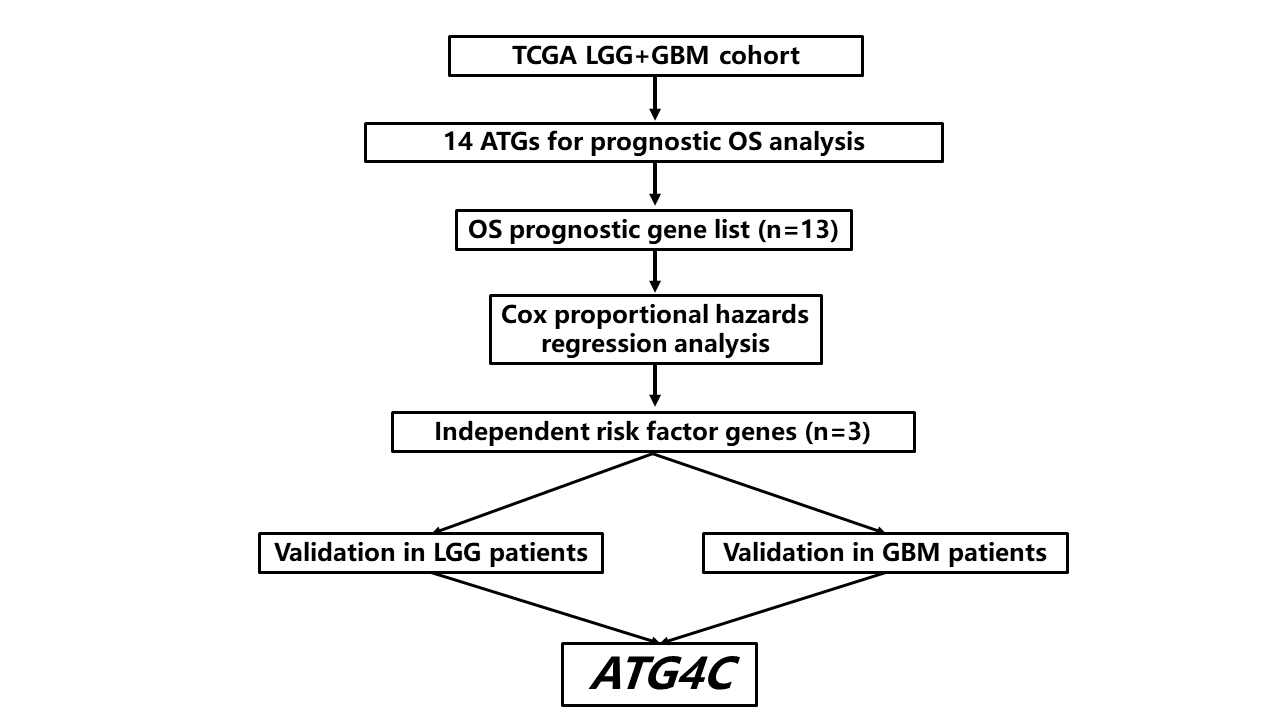

Supplement: Supplementary file 1 — Figure S1. Flow chart of procedures for screening of ATGs associated with outcomes of glioma patients. (TIF 90 kb) [file 13046_2019_1287_MOESM1_ESM.tif]

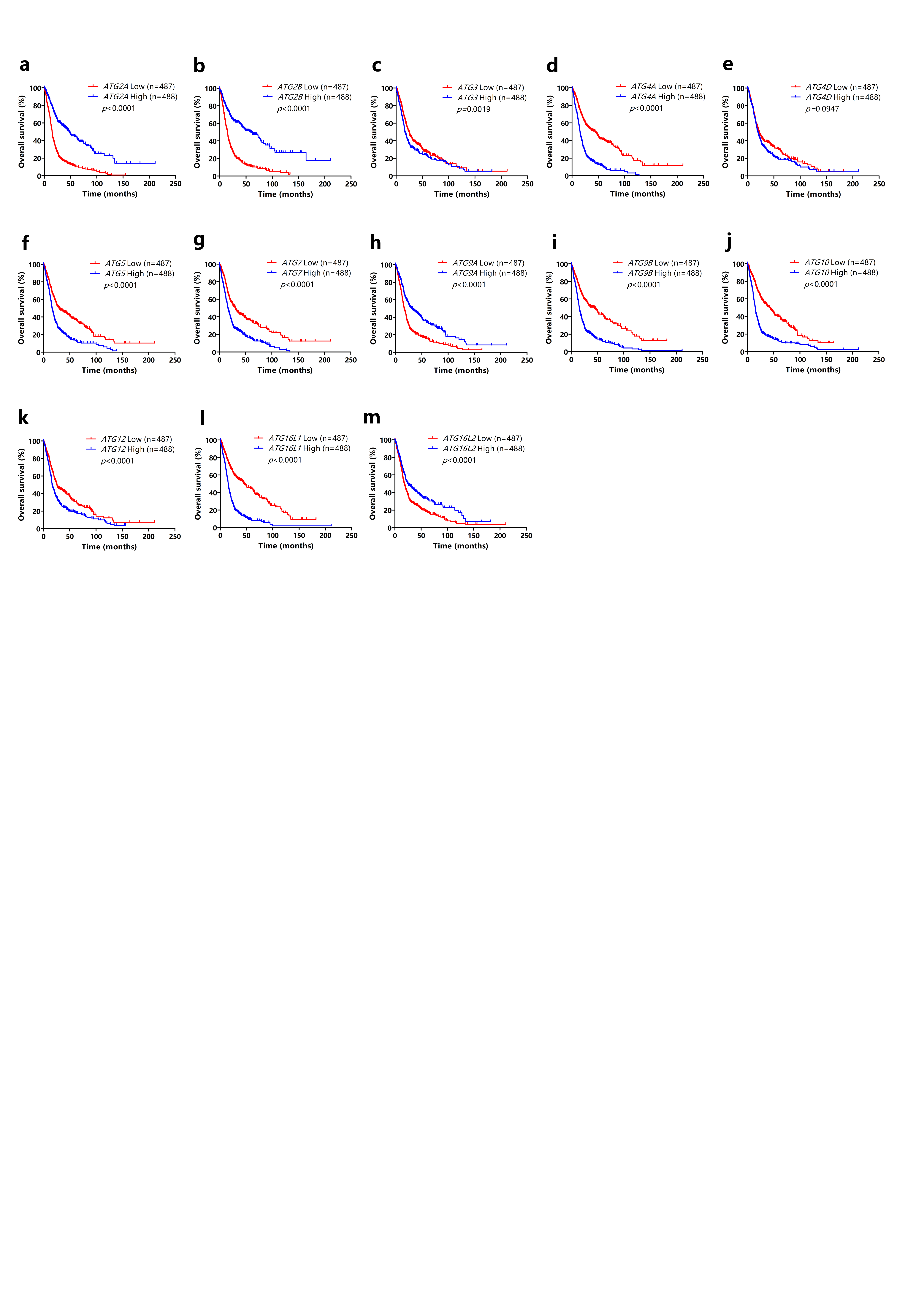

Supplement: Supplementary file 2 — Figure S2. Survival analysis of glioma patients based on expression of ATGs. (a-m) Kaplan-Meier analysis for OS in gliomas patients. (TIF 2897 kb) [file 13046_2019_1287_MOESM2_ESM.tif]

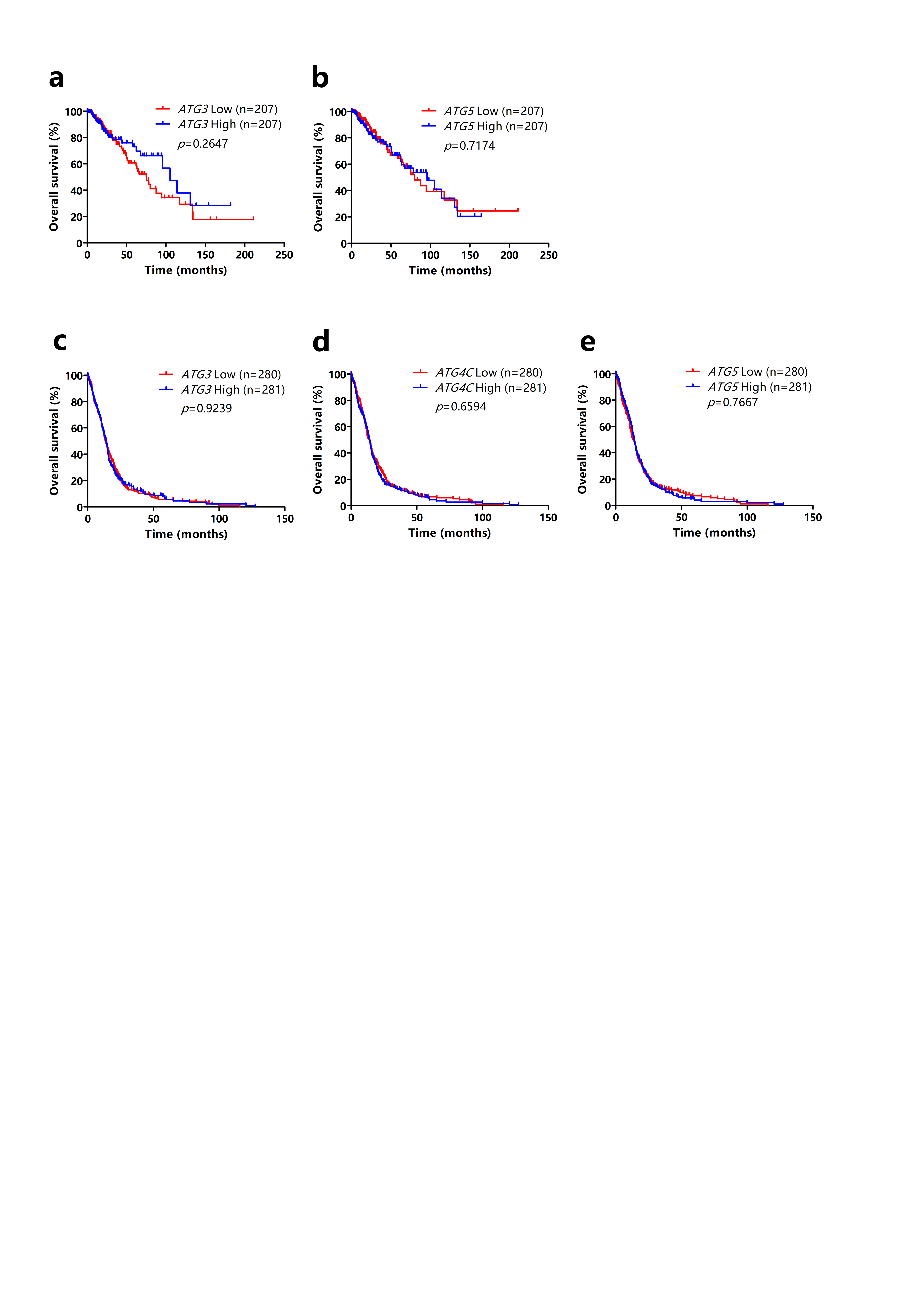

Supplement: Supplementary file 3 — Figure S3. Survival analysis of LGG based on expression of ATGs. (TIF 2249 kb) [file 13046_2019_1287_MOESM3_ESM.tif]

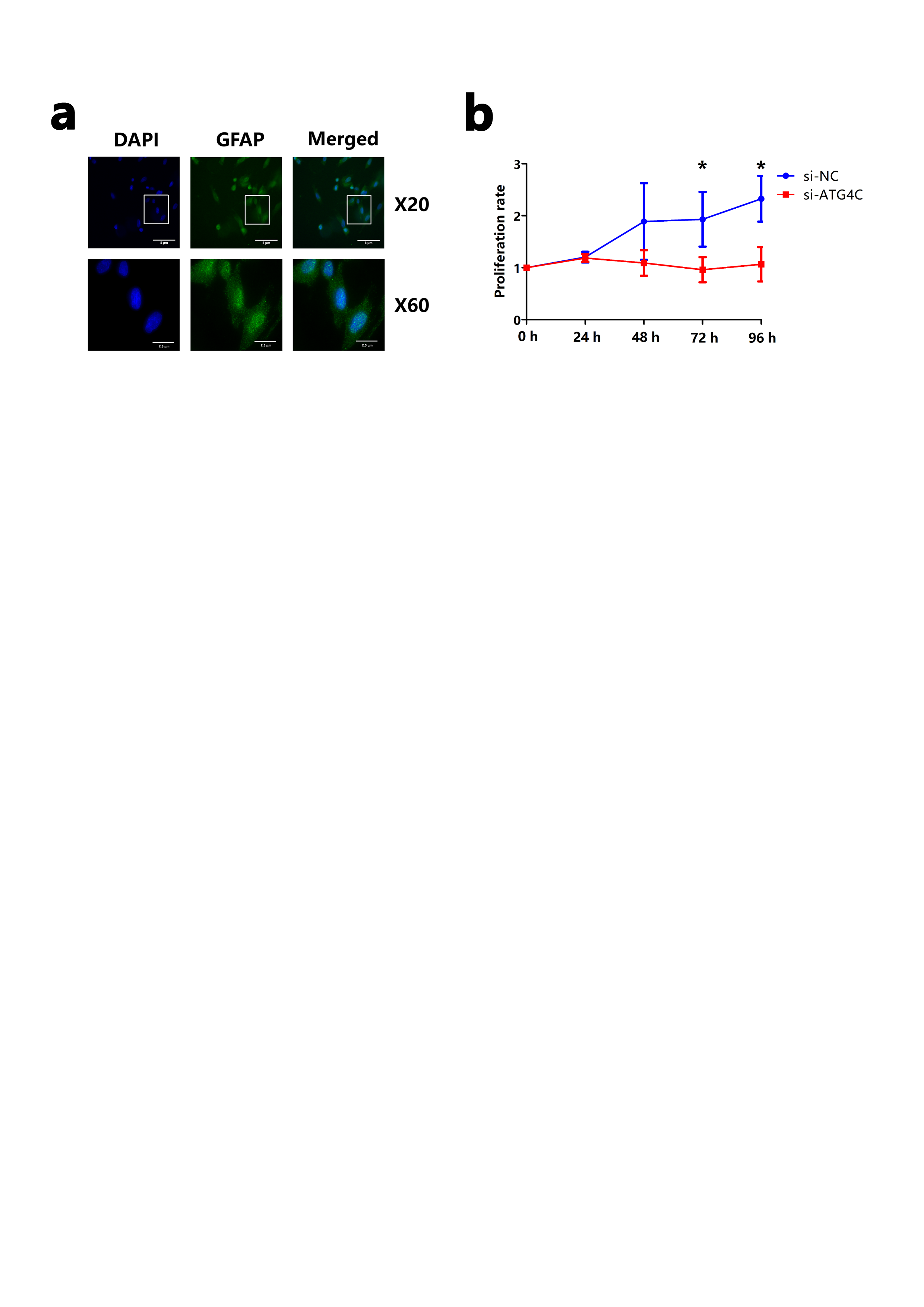

Supplement: Supplementary file 4 — Figure S4. Knockdown of ATG4C suppressed cell growth in glioblastoma primary cells. a The expression of GFAP in glioblastoma primary cells. b ATG4C knockdown suppressed the proliferation of primarily cultured glioblastoma cells. (TIF 952 kb) [file 13046_2019_1287_MOESM4_ESM.tif]
