# Supplementary material for: Knockdown ATG4C inhibits gliomas progression and promotes temozolomide chemosensitivity by suppressing autophagic flux
Source: J Exp Clin Cancer Res. 2019 Jul 10;38:298. doi: 10.1186/s13046-019-1287-8 (PMC6617611; doi:10.1186/s13046-019-1287-8)
Supplement: Supplementary file 5 — Table S1. Sequences of primers used for RT-qPCR. (DOCX 14 kb) [file 13046_2019_1287_MOESM5_ESM.docx]

Table S1 Sequences of primers used for RT-qPCR

| Gene | Forward (5’-3’) | Reverse (5’-3’) |
| --- | --- | --- |
| *GAPDH* | CTGCACCACCAACTGCTTAG | AGGTCCACCACTGACACGTT |
| *ATG12* | ACCCATTGCTCCTACTTGTTACTA | TTTCTGCCTGGTGGACTGC |
| *ATG5* | ATCAGGTTTGGTGGAGGCA | GGTTTAATGATGGCAGTGGAGG |
| *ATG7* | CCAAGGTCAAAGGACGAAGAT | GTACGGTCACGGAAGCAAAC |
| *ATG16L1* | TGGACAAGTTCTCAAAGAAGCTG | CCTCAGTGCGACCAGTGAT |
| *ATG4C* | TAGAGGATCACGTAATTGCAGGA | TAGAGGATCACGTAATTGCAGGA |
