# Supplementary material for: Knockdown ATG4C inhibits gliomas progression and promotes temozolomide chemosensitivity by suppressing autophagic flux
Source: J Exp Clin Cancer Res. 2019 Jul 10;38:298. doi: 10.1186/s13046-019-1287-8 (PMC6617611; doi:10.1186/s13046-019-1287-8)
Supplement: Supplementary file 6 — Table S2. Cox proportional hazards regression analysis for OS in LGG patients. (DOCX 17 kb) [file 13046_2019_1287_MOESM6_ESM.docx]

| Variable | Univariate analysis | |  | Multivariate analysis | |
| --- | --- | --- | --- | --- | --- |
|  | HR (95% CI) | p value |  | HR (95% CI) | p value |
| IDH | 0.16 (0.10-0.26) | 7.03×10^-14^ |  | 0.37 (0.20-0.67) | 0.001 |
| Grade Ⅲ vs Grade Ⅱ | 3.38 (2.04-5.60) | 2.27×10^-6^ |  | 2.21 (1.28-3.80) | 0.004 |
| Age | 1.07 (1.05-1.09) | 5.08×10^-12^ |  | 1.06 (1.04-1.09) | 4.66×10^-10^ |
| Gender | 0.89 (0.57-1.40) | 0.62 |  | 1.00 (0.62-1.61) | 0.99 |
| *ATG3* | 0.41 (0.17-0.99) | 0.05 |  | 0.32 (0.12-0.83) | 0.02 |
| *ATG4C* | 2.37 (1.53-3.68) | 1.15×10^-4^ |  | 2.95 (1.64-5.32) | 3.05×10^-1^ |
| *ATG5* | 0.85 (0.35-2.07) | 0.73 |  | 0.96 (0.41-2.27) | 0.93 |

Table S2. Cox proportional hazards regression analysis for OS in LGG patients
