# Supplementary material for: Knockdown ATG4C inhibits gliomas progression and promotes temozolomide chemosensitivity by suppressing autophagic flux
Source: J Exp Clin Cancer Res. 2019 Jul 10;38:298. doi: 10.1186/s13046-019-1287-8 (PMC6617611; doi:10.1186/s13046-019-1287-8)
Supplement: Supplementary file 7 — Table S3. Cox proportional hazards regression analysis for RFS in LGG patients. (DOCX 18 kb) [file 13046_2019_1287_MOESM7_ESM.docx]

| Variable | Univariate analysis | |  | Multivariate analysis | |
| --- | --- | --- | --- | --- | --- |
|  | HR (95% CI) | p value |  | HR (95% CI) | p value |
| IDH | 0.18 (0.11-0.29) | 6.77×10^-12^ |  | 0.26 (0.15-0.47) | 6.11×10^-6^ |
| Grade Ⅲ vs Grade Ⅱ | 1.76 (1.16-2.71) | 0.01 |  | 1.20 (0.75-1.92) | 0.45 |
| Age | 1.02 (1.00-1.03) | 0.07 |  | 1.01 (0.99-1.02) | 0.32 |
| Gender | 0.81 (0.54-1.22) | 0.31 |  | 0.89 (0.58-1.36) | 0.59 |
| *ATG3* | 0.76 (0.33-1.74) | 0.51 |  | 0.61 (0.24-1.54) | 0.30 |
| *ATG4C* | 2.05 (1.39-3.03) | 2.93×10^-4^ |  | 1.83 (1.14-2.92) | 0.01 |
| *ATG5* | 0.76 (0.33-1.76) | 0.53 |  | 0.64 (0.30-1.35) | 0.24 |

Table S3. Cox proportional hazards regression analysis for RFS in LGG patients
